# mHealth smoking cessation intervention among high school students: 3-month primary outcome findings from a randomized controlled trial

**DOI:** 10.1371/journal.pone.0229411

**Published:** 2020-03-06

**Authors:** Ulrika Müssener, Catharina Linderoth, Kristin Thomas, Marcus Bendtsen

**Affiliations:** Department of Health, Medicine and Caring Sciences, Faculty of Medicine and Health, Linköping University, Linköping, Sweden; Global Public Health, New York University, UNITED STATES

## Abstract

**Background:**

Smoking among adolescents remains a global public health issue as youth continue to maintain high prevalence rates. The evidence for the efficacy of text messaging interventions to reduce smoking behavior is well established, yet there is still a need for studies targeting high school students. The aim of the study was to determine the effectiveness of a text-based smoking cessation intervention among high school students in Sweden.

**Methods:**

The study was a two-arm randomized trial conducted from January 10 2018 to January 11 2019, data were analysed from April 12 2019 to May 21 2019. Inclusion criteria were high school students who were daily or weekly smokers willing to attempt to quit smoking and owned a mobile phone. The study invited all students at 630 high schools units throughout Sweden. The intervention group received text messages based on components of effective smoking cessation interventions for 12 weeks. The control group were offered treatment as usual. The primary outcomes were self-reported prolonged abstinence (not having smoked more than 5 cigarettes over the last 8 weeks) and 4-week point prevalence of smoking abstinence.

**Findings:**

A total of 535 participants, with a median age of 17 (IQR 16–18), were randomized into the study; 276 (164 [59.4%] women) were allocated to the intervention and 259 (162 [62.5%] women) to the control group. The outcomes of the trial were analyzed on a total of 212 (76.8%) participants in the intervention group and 201 (77.6%) participants in the control group. Prolonged abstinence at the 3-month follow-up was reported by 49 (23.1%) individuals in the intervention group and 39 (19.4%) individuals in the control group (adjusted OR, 1.21; 95% CI, 0.73–2.01; P value, .46). Four-week point prevalence of complete smoking cessation was reported by 53 (25.0%) individuals in the intervention group and 31 (15.4%) individuals in the control group (adjusted OR, 1.87; 95% CI, 1.12–3.17; P value, .018).

**Conclusions:**

Estimates of 4-week point prevalence of complete cessation was 10 percentage points higher in the group that were given access to the intervention compared to the control. Findings provide confirmation that text messaging-based smoking cessation programs can affect quit rates among adolescents.

**Trial registration:**

ISRCTN15396225; registration date October 13, 2017, https://trialsjournal.biomedcentral.com/articles/10.1186/s13063-018-3028-2.

## Introduction

Although the major burden of disease caused by smoking falls on adult populations, smoking is still among the leading causes of premature death globally [1,2]. Reductions in smoking prevalence have been observed, but smoking among adolescents remains a global public health issue as youth continue to maintain high prevalence rates [3,4]. Sweden have the lowest smoking prevalence within high-income countries, still smoking rates among this age demographic have yet to decline [5]. Around 25% of high school students were smokers in 2016, of whom a third were daily smokers. The prevalence of daily smoking among young people between 16 and 29 years of age has been stable in the last 5 years in Sweden at approximately 11% to 13% among women and 7% to 10% among men [5–7].

Unhealthy lifestyle behaviors, such as smoking, tend to be established in early childhood and adolescence and track into adulthood [8–10], therefore prevention efforts and smoking cessation interventions that are effective for adolescents are vital [3,11]. Figures show that a majority, around two thirds of high school smokers, want to quit [7]. Younger age groups have the highest quit attempt rates, which decline with age [12], indicating that adolescents are a ripe audience for assisting in smoking cessation [13]. However, young people are less likely to use cessation interventions, compared with their older counterparts [14–16]; only about one in ten seek or gain access to evidence-based interventions [17]. The lack of tailored cessation interventions for this age demographic has since long been cited as a major reason for this underutilization [15].

A promising direction for reaching and engaging adolescents in smoking cessation interventions is their use of mobile phones. Mobile phone interventions may offer a cost-effective and readily available avenue to provide personalized support adolescents. This intervention medium is especially suited for this group for several reasons. First, mobile phone ownership and use are nearly universal. In addition, mobile phone interventions can be utilized for little cost, and with high fidelity, and can be utilized without the possible stigma of having to inform friends and family members of their need of quit smoking. These interventions can be provided to individuals within their own environment and delivered in real-time [17–21].

Evidence of the efficacy of text messaging interventions to reduce smoking behavior across diverse adult populations is well established and supported by results from meta-analysis [22,23], systematic reviews [24], and of several randomized controlled trials (RCTs) [20,25–29]. Indeed, text message-based interventions have been identified as one of the most affordable interventions for global tobacco control and has been endorsed by the World Health Organization [11].

Even though schools provide convenient access to adolescents, little effort has been made in the school setting to offer support to students who wish to quit smoking; rather the emphasis has been on prevention of uptake [30]. More recently, researchers have begun to test text messaging intervention programs on samples that include adolescents and young adults. In a review by Mason et al [18], a total of 14 RCTs were included in a meta-analysis of the efficacy of text messaging intervention for tobacco and alcohol cessation with adolescents and young adult populations, ages 12 to 29. Eleven studies targeted tobacco use (*n* = 10,573) and three focused on alcohol (*n* = 79) for a total of 10,652 research participants. No study was exclusive to adolescents. The studies included in the meta-analysis were heterogeneous and broadly defined, comprising any intervention that included a component where text messages were sent to participants’ mobile phones. Results yielded a summary effect size of 0.25, indicating that in general, text interventions have a positive effect on reducing substance use behaviors [18]. Research is ongoing, e.g. a study protocol by Prokhorov describes a study that aims to outline the rationale and design of a randomized trial comparing the effects of different text message structures among college students [16].

Despite the fact that: 1) all high schools in Sweden are bound by law to offer a smoke-free environment, 2) young adults are the heaviest users of technology, 3) the promise of text-based interventions for quit smoking is positive, there are no effective nor evidence based text messaging interventions that target high school students. This report contains the analyses of primary outcomes from a randomized trial which aimed to estimate the effects of a text message-based smoking cessation intervention targeting high school students in Sweden.

## Methods

### Study design and participants

Nicotine Exit Junior (NEXit Junior) was a two-arm randomized controlled trial of a text messaging smoking cessation intervention in which participants were randomized to an intervention group (smoking cessation program) or a control group (treatment as usual). The study was undertaken simultaneously in 630 high school units in Sweden. Recruitment was executed by paper advertising (posters and leaflets), digital advertising (e-mail, school website, and/or app if available) and by school staff (teachers, mentors, and/or school health centres). Recruitment was completed over a one year period, ending in January 2019 [31]. The study was approved by the Regional Ethical Committee in Linköping, Sweden (Dnr 2017/388-31).

Eligible participants were high school students who were daily or weekly smokers, willing to attempt to quit smoking, and who owned a mobile phone. Participants registered their interest by sending a text message to a dedicated telephone number and received immediate confirmation. Participants provided consent by pressing a hyperlink in the confirmation message and were subsequently transferred to a baseline assessment questionnaire [31]. Follow-up began at the beginning of May 2018 and ended in April 2019.

### Randomization and blinding

After completing the baseline questionnaire, participants were randomized to the intervention group or the control group. A text message was sent to each participant to inform them of allocation. The message sent to participants allocated to the control group included information on how to contact the national smoking cessation help line offering smoking cessation support to youth. No additional prompts, reminders, or information were given to the control group during the study. Randomization was fully computerized and automated with equal allocation, using no blocks or strata, using Java’s built in random number generator (java.util.Random). The final allocation sequence was tested with a runs test to identify dependence between elements in the sequence. No dependence was found; thus allocation was mutually independent.

### Interventions

The intervention under trial was based on a similar intervention that was previously developed targeting an adult group [29]. It was then further developed, tailored and targeted for adolescents. For example, information on smoking harm during pregnancy and birth was removed and fewer tips to avoid weight gain was given. The intervention was based on existing evidence-based practice and included key elements from previous text message-based interventions and Internet-based interventions [23–28, 32, 33]. The intervention was developed using formative methods [34–37], and behavior change technique analysis [38]. The content of the text messages comprised information about the health risks of smoking, tips on behavior change strategies, and activities. The program included the following elements: making a public declaration about quitting, encouraging asking for support from family and friends, distraction techniques and tips to avoid weight gain, tips to cope with cravings and to avoid smoking triggers, and how to distract one’s mind from smoking [38].

The intervention consists of a 12-week automated program with a total of 121 text messages. The program starts with two to four messages per day during the first two weeks, two messages per day during week three, one message per day during weeks four to seven, and during weeks 8–12 an average of five messages per week is sent [31].

### Outcomes

The first primary outcome was prolonged abstinence, defined as not having smoked more than five cigarettes in the past 8 weeks. This follows the Russel standard for smoking interventions, allowing for a grace period of 4 weeks (12-week program) [39]. The second primary outcome was 4-week point prevalence of not having smoked a single cigarette at the time of follow up, which aims to identify delayed effects of the intervention, a recommended outcome measure by the Society for Research on Nicotine and Tobacco [40].

Follow-up responses were collected by sending a text message to all participants’ 3-months after randomization. The text message included a hyperlink to a web questionnaire. Two reminders were sent 2 days apart. Those who had not responded after the third message were called by phone (a maximum of 10 attempts per participant).

### Sample size

Based on our earlier research [29], we expected a difference of approximately 10% in cessation rates between the two groups. To achieve 80% power with a significance level of .05 (two-sided) and correction for continuity, a sample size of 195 individuals were needed in each group. Assuming 30% attrition in the follow-up measurements, the number needed in each group is 279, with a total required sample size of 558.

### Statistical analysis

The data analysis followed the prespecified statistical analysis plan published in the trial protocol [31]. The intention-to-treat analysis strategy included all participants with follow-up data in their groups as randomized, and sensitivity analyses included all randomized participants to explore different assumptions about any missing data.

The two primary binary outcomes (8-week prolonged abstinence and 4-week point prevalence of complete smoking cessation) were analyzed by logistic regression, and the results presented as odds ratios and 95% confidence intervals (CIs). All regression analyses were adjusted for the following baseline variables: gender, duration of smoking, average number of cigarettes smoked weekly, severity of dependence as measured by Fagerström’s Nicotine Dependence Scale, and amount of snuff used at baseline. Effect modification analyses were completed with the following potential effect modifiers measured at baseline: gender, average number of cigarettes smoked weekly, amount of snuff used weekly, and severity of dependence. Each effect modification analysis was performed by comparing adjusted logistic regression models, excluding and including the interaction parameter using the likelihood ratio test.

### Sensitivity analyses

Attrition and sensitivity analyses explored the effects of departures from the missing at random (MAR) assumption in the main analysis. We used the number of attempts required to collect follow-up data to explore the plausibility of the MAR assumption by exploring the association between quitting and the number of follow-up attempts needed.

All tests were performed two-sided at the .05 significance level. Data were analyzed from April to May 2019. R version 3.4.2 was used for all analyses.

## Results

**Fig 1:**
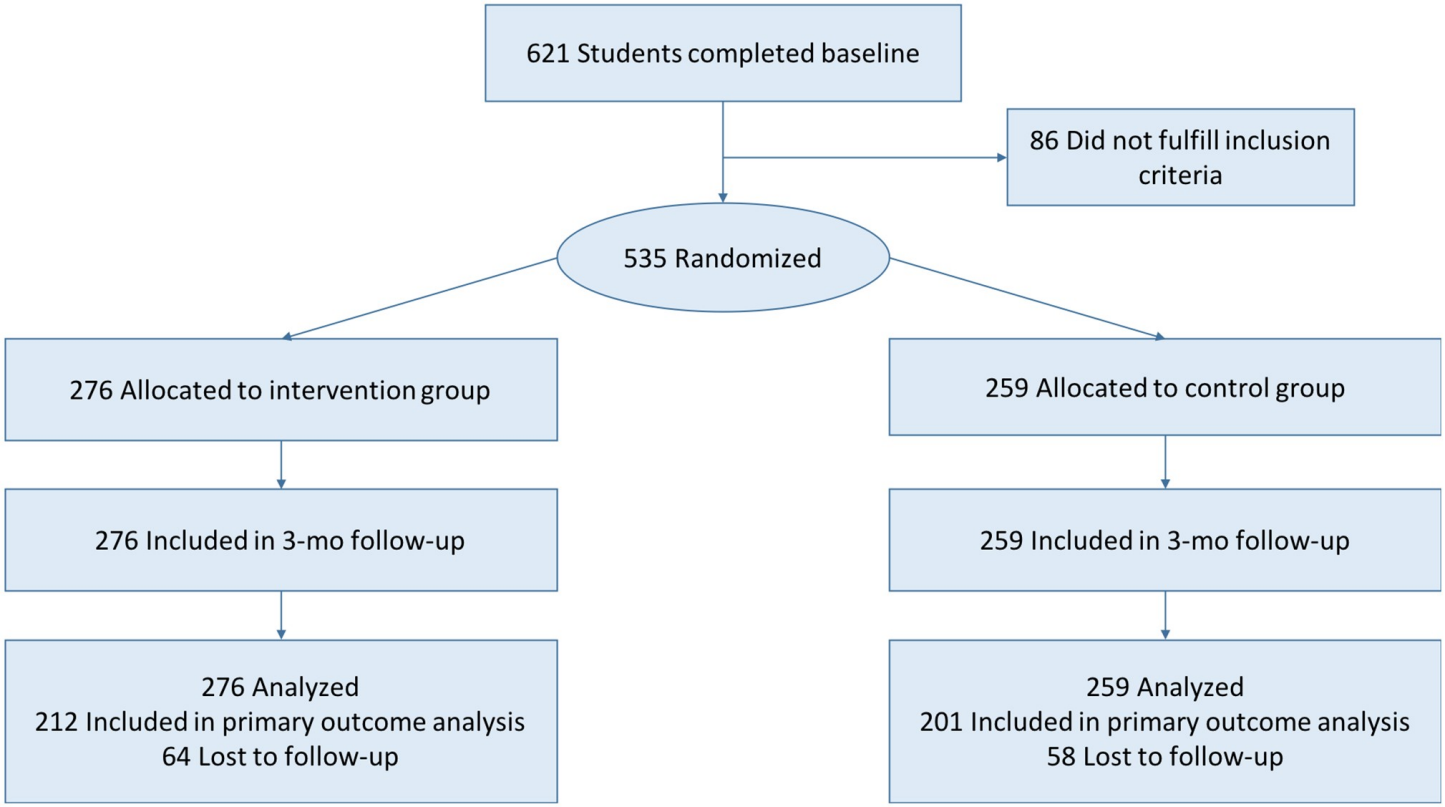
CONSORT flowchart.

A total of 535 students were randomized: 276 (51.6%) to the NEXit Junior intervention group and 259 (48.4%) to the control group, please see Fig 1. There were no statistically significant differences between the groups with respect to gender, age, recruitment strategy, or smoking variables at baseline (Table 1).

**Table 1:** Baseline characteristics of participants in the intervention and control groups.

Variables	Intervention (n = 276)	Control (n = 259)	P value
Gender, No. (%)			.91^a^
Female	164 (59.4)	162 (62.5)	
Male	102 (37)	90 (34.7)	
Other	3 (1.1)	2 (0.8)	
Decline to answer	4 (1.4)	2 (0.8)	
Do not know	3 (1.1)	3 (1.2)	
Age, median (IQR), y	17 (16–18)	17 (16–18)	.69^b^
Duration of smoking, median (IQR), y	3 (2–5)	3 (2–5)	.22^b^
No, cigarettes per week, median (IQR), y	60 (35–84)	70 (42–105)	.061^b^
Using snus, No. (%)	84 (30.4)	74 (28.6)	.71^c^
Fagerström Nicotine Dependence Scale, median (IQR)	4 (2–6)	4 (3–5.5)	.26^b^
Quit attempts, median (IQR)	2 (1–4)	2 (1–4)	.85^b^
Previous use of nicotine replacement therapies, median (IQR)	0 (0–1)	0 (0–1)	.96^b^
Cessation counseling experience, No. (%)			.21^c^
No	250 (90.6)	226 (87.3)	
Yes, previously	13 (4.7)	22 (8.5)	
Yes, currently	13 (4.7)	11 (4.2)	
Recruitment strategy, No. (%)			.46^a^
Poster	71 (25.7)	80 (30.9)	
Homepage	49 (17.8)	37 (14.3)	
Student health center	45 (16.3)	44 (17.0)	
Staff	34 (12.3)	34 (13.1)	
School’s mobile app	33 (12.0)	32 (12.4)	
Friend	21 (7.6)	10 (3.9)	
Flyer	10 (3.6)	5 (1.9)	
E-mail	4 (1.4)	5 (1.9)	
Other	9 (3.3)	12 (4.6)	

Abbreviation: IQR, interquartile range (lower [25^th^ percentile] to upper [75^th^ percentile] quartiles)

^a^ P value by Fisher’s exact test

^b^ P value by Wilcoxon rank sum test

^c^ P value by chi-square test

### Outcome analysis

The outcomes of the trial were analyzed on a total of 212 (76.8%) participants in the intervention group and 201 (77.6%) participants in the control group. Among those analyzed, 49 (23.1%) individuals in the intervention group and 39 (19.4%) individuals in the control group reported prolonged abstinence at the 3-month follow-up (having smoked less than 5 cigarettes the past 8 weeks). Adjusted logistic regression did not identify a statistically significant difference between the two groups (adjusted OR, 1.21; 95% CI, 0.73–2.01; P value, .46). Four-week point prevalence of complete smoking cessation was reported by 53 (25%) of analyzed intervention group participants, and 31 (15.4%) of analyzed control group participants. Adjusted logistic regression did identify a statistically significant difference between the two groups (adjusted OR, 1.87; 95% CI, 1.12–3.17; P value, .018), please see Table 2. Regression models including baseline characteristics as effect modifiers did not show any evidence of modification (likelihood ratio tests gave P values from 0.09 to 0.60).

**Table 2:** Comparing outcomes between intervention and control.

	No. (%)		Intervention vs. Control, Odds ratio (95% CI)			
Variables	Intervention (n = 212)	Control (n = 201)	Unadjusted	P value	Adjusted^a^	P value
Self-reported prolonged abstinence	49 (23.1)	39 (19.4)	1.25 (0.78–2.01)	.36	1.21 (0.73–2.01)	.46
Self-reported 4-wk point prevalence of complete smoking cessation	53 (25.0)	31 (15.4)	1.83 (1.12–3.02)	.017	1.87 (1.12–3.17)	.018

^a^ Adjusted for gender, number of years smoked, number of cigarettes smoked weekly, Fagerström Nicotine Dependence Scale (score), and amount of snus used at baseline. Gender categories “Other”, “Declined to answer” and “Do not know” were pooled.

### Attrition and sensitivity analysis

Departures from the MAR assumption were investigated by attrition and sensitivity analyses. There were no statistically significant differences among those who completed follow-up and those who did not with respect to baseline variables. Assuming that all follow-up non-responders continued to be smokers did not dramatically change ORs for either outcome (prolonged abstinence adjusted OR, 1.20 and point prevalence adjusted OR, 1.74).

To further explore the MAR assumption, we created a dummy variable representing the number of attempts required to collect follow-up data. Responders to the initial SMS required 1 attempt, responders to the second SMS required 2 attempts, responders to the third SMS required 3 attempts, responders to the first phone call required 4 attempts, and so on up to attempt 13 (ie., responded to the 10^th^ phone call attempt). Two logistic regression models were created for each of the primary outcomes (8-week prolonged abstinence and 4-week point prevalence), where the outcome was regressed against the attempts dummy variable alone in one model, and group allocation and an interaction term between the attempts and group allocation were added in the second model. The models with only the attempts variable indicated significant differences between early and late responders: prolonged abstinence (coefficient estimate (log scale) 0.11; 95% CI, 0.02–0.20; P value, .014) and point prevalence (coefficient estimate (log scale) 0.10; 95% CI, 0.01–0.19, P value, .032), with late responders more likely to be non-smokers. The models adjusting for group allocation and including an interaction between attempts and group allocation did however not reveal any statistically significant differences (P values of .065 and .299 respectively), thus the systematic difference between early and late responders was not stronger in either group.

## Discussion

To address the lack of smoking cessation programs available to adolescents, we developed and estimated the effect of a text messaging–based smoking cessation program. The findings in this report indicate that while it may take longer for high school students to quit smoking completely, estimates of 4-week point prevalence of complete cessation was 10 percentage points higher in the group that were given access to the intervention compared to the control. Considering that participants were recruited from a non-treatment seeking population, it is quite astonishing that an OR of 1.87 can be attributed to a 12-week text message-based intervention. Furthermore, the cost of routinely offering this type of intervention to all high school students in Sweden is virtually nonexistent in comparison with the long-term health benefits that this young group of individuals may gain from quitting.

Major strengths of this study include that the fully automated system does not involve personal interactions, as used in previous research targeting a young adult population [13,18] and adults [22–25]. Hence, the intervention can be made available nationwide at very low cost, requiring little or no resources from staff at participating high schools. Since the mHealth intervention offers an alternative to face-to-face interventions, it may prevent stigma and embarrassment about seeking help, which are identified barriers for help-seeking among adolescents [18]. A potential limitation of the study is that follow-up was assessed by self-reported measures and was not biochemically verified. But, any bias in reporting may be assumed to be equal in both the intervention group and the control group. Furthermore, based on recommendations by The Society for Research on Nicotine and Tobacco, in population-based studies with limited face-to-face contact, it is neither required, nor desirable to use biochemical verification [40]. This report contains analyses of short-term effects of the program, and future reports will investigate if the effects persist, however it is important to note that we do not expect any long term effects from a 12-week program if there are no short term effects, thus the reported findings are encouraging. Also, we have no reason to suspect that participants in the intervention group are more likely than participants in the control group to start smoking again, thus over time there should be fewer smokers among those who were given access to the intervention.

Considering the well-known challenges of conducting trials with this target group [41,42], we expected a loss-to-follow-up of approximately 30%. We were however able to retain more participants than expected, resulting in an attrition rate of 23.2%. Attrition analyses did not detect any differences between responders and non-responders with respect to baseline variables, and assuming that all non-responders continued smoking did not change the outcome of the analyses drastically. There was however a systematic trend indicating that late responders were more likely to have quit smoking that early responders. There was however no evidence that this trend was stronger in either group. If we believe that non-responders are more like late responders than earlier responders, then the MAR assumption should at least to some degree be questioned in the analysis of the data from this trial.

A Cochrane review [3] from 2017 included 28 trials, targeting young people aged under 20 years, to evaluate the effectiveness of strategies that help young people to stop smoking tobacco. The studies included were of mixed quality and looked at various methods for stopping smoking, including one-to-one counselling, counselling as part of a group, methods using computers or text messaging, or a combination of these. The review showed insufficient evidence to recommend a specific method of intervention for young people and that there continues to be a need for well-designed, adequately powered, randomized controlled trials of interventions for this population of smokers [3]. Since earlier efforts have focused on smoking prevention [30], little effort has gone into finding effective ways of systematically helping students who wish to quit smoking.

There is increasing evidence that the use of text messaging can help people quit smoking. Results from our present study are in line with previous research [18], which demonstrated the potential of text message-based interventions to reach a high proportion of young smokers. In our previous study [29] targeting university students, the text-message intervention almost doubled the rate of prolonged abstinence at the 4-month follow up.

Our findings suggests that NEXit Junior is acceptable and effective for this younger target group, who otherwise do not access evidence-based smoking cessation support: the median number of uses of nicotine replacement therapies was 0 (IQR 0–1) and a majority (89%) had not had any cessation counseling experience.

In summary, several biological, psychological, and social transitions that occur during adolescent are essential for a young person’s later-life trajectory. Adolescence is a sensitive period in terms of taking up smoking, which in turn has long-term consequences for smoking in adulthood. Capturing the attention of young adults can be challenging. Results from the NEXit Junior trial demonstrated a positive effect on smoking cessation of an intervention consisting of text messages only, which has been designed to have extensive reach among young smokers.

## Supporting information

S1 File (PDF)

S1 Checklist CONSORT checklist. (DOC)
